# Combination treatment with n-3 polyunsaturated fatty acids and ursodeoxycholic acid dissolves cholesterol gallstones in mice

**DOI:** 10.1038/s41598-019-49095-z

**Published:** 2019-09-04

**Authors:** Sung Ill Jang, Sungsoon Fang, Kwang Pyo Kim, Younhee Ko, Hyoseon Kim, Jieun Oh, Ga Young Hong, Su Yeon Lee, Joon Mee Kim, Ilkoo Noh, Dong Ki Lee

**Affiliations:** 1 Department of Internal Medicine, Gangnam Severance Hospital, Yonsei University College of Medicine, Seoul, Republic of Korea; 2 Severance Biomedical Science Institute, BK21 Plus Project for Medical Science, Gangnam Severance Hospital, Yonsei University College of Medicine, Seoul, Republic of Korea; 3 Department of Applied Chemistry College of Applied Sciences, Kyung Hee University, Yong-in City, Republic of Korea; 4 Division of Biomedical Engineering, Hankuk University of Foreign Studies, Yongin, Republic of Korea; 5 Department of Biological Sciences, Seoul National University, Seoul, Republic of Korea; 6 Department of Pathology, Inha University College of Medicine, Incheon, Republic of Korea; 7 Department of Chemical and Biomolecular Engineering, Korea Advanced Institute of Science and Technology, Daejeon, Republic of Korea

**Keywords:** Cholelithiasis, Cholelithiasis

## Abstract

The increasing prevalence of cholesterol gallstone disease places an economic burden on the healthcare system. To identify novel therapeutics, we assessed the effects of n-3 polyunsaturated fatty acids (PUFA) in combination with UDCA in a mouse model of cholesterol gallstones. Gallstone dissolution, gallbladder wall thickness, mucin gene expression in the gallbladder, and levels of phospholipids, cholesterol, and bile acids in bile and serum were analysed. RNA was extracted from the liver for mRNA sequencing and gene expression profiling. Combination treatment resulted in greater gallstone dissolution compared with the control group, and PUFA and combination treatments reduced the thickness of the gallbladder wall. Expression levels of mucin genes were significantly lower in the UDCA, PUFA, and combination groups. Transcriptome analyses revealed that combination treatment modulated hepatic lipid metabolism. The PUFA and combination groups showed elevated bile phospholipid and bile acid levels and a lower cholesterol saturation index. Combination treatment with PUFA and UDCA dissolves cholesterol gallstones in mice by decreasing mucin production, increasing levels of phospholipids and bile acids in bile, and decreasing cholesterol saturation. Further studies of the therapeutic effects of combination PUFA and UDCA treatment in patients with cholesterol gallstones are warranted.

## Introduction

Gallstone disease is an important health problem with gastrointestinal sequelae that frequently leads to admission to the hospital^1,2^ and has a mortality rate of 0.6%^1^. In the United States, patients with gallstone disease have an increased overall mortality rate, particularly due to cardiovascular disease and cancer^3^. As the incidence of gallstone disease increases, so does the incidence of complications such as gallstone-related pancreatitis^4^.

Cholesterol gallstone disease is associated with an increased concentration of cholesterol relative to that of bile salts and phospholipids. When the amounts of biliary lipids, inorganic salts, and/or organic salts in bile acids exceed maximum solubility, the acids crystallize through nucleation and eventually form gallstones^5^. Westernized diets, which are high in calories, cholesterol, saturated fatty acids, carbohydrates, and protein and low in dietary fiber, promote gallstone formation^6,7^. For instance, Canadian Eskimos who consume a Western-style diet have an increased prevalence of complications due to cholesterol gallstone disease^8^. Furthermore, the number of patients with cholesterol or mixed gallstones has rapidly increased in Asia as the Asian diet has become increasingly westernized^9,10^.

Gallstones are present in 10–20% of the general population, although 80% are asymptomatic^11^. However, serious symptoms and complications that require treatment develop in 1–2% of patients with asymptomatic gallstones annually^12^. Surgical treatments such as cholecystectomy are standard for symptomatic gallstone disease, but there are few medical treatment options. Although laparoscopic cholecystectomy is safer than open cholecystectomy, it has serious risks^13,14^. The prevalence of laparoscopic cholecystectomy among gallstone patients is 1.6–12%, and its complication rate can reach 1.2%, with bile duct damage being the major cause of complications^13^. Because patients with gallstones who undergo surgical treatment can experience chronic biliary pain and gallstone-related complications^15^, effective medical treatments are required. Since the introduction of chenodeoxycholid acid and ursodeoxycholic acid (UDCA) for the treatment of gallstones in the 1970s and 1980s, respectively, treatment with oral solvents has replaced high-risk open cholecystectomy^16,17^.

Epidemiological studies have investigated the potential therapeutic effects of omega-3 polyunsaturated fatty acids (PUFAs) against cholesterol gallstones. For instance, Eskimos, who have a diet rich in fish oil (which contains a high level of omega-3 PUFAs and has two major components, eicosapentaenoic acid [EPA, C20:5n-3] and docosahexaenoic acid [DHA, C22,6n-3])^18^, have a lower prevalence of cholesterol gallstones than that of Western populations^19^. In this study, we evaluated the therapeutic effects of PUFA, UDCA, and the combination of PUFA and UDCA in a mouse model of cholesterol gallstones.

## Results

### Gallstone formation

**Figure 1:**
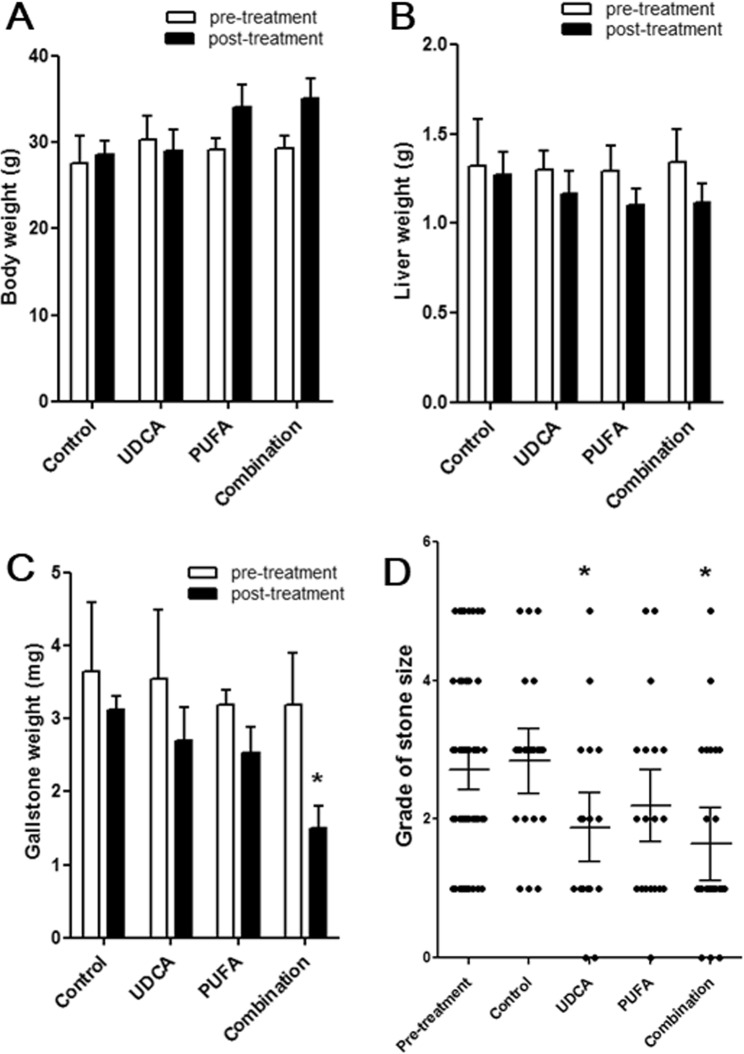
Body weight, liver weight, and gallbladder stone weight and size before and after treatments. Body weight, liver weight, and gallbladder stone weight and size were checked during the pre-treatment (after the feeding with lithogenic diet for 8 weeks) and post-treatment stages (after administration of the therapeutic agent with a regular diet for 12 weeks) in the four groups (control, ursodeoxycholic acid [UDCA], polyunsaturated fatty acids [PUFA], and combination group). The variables were evaluated in 15 mice in each group during the pre-treatment stage, and in 25 mice in each group during the post-treatment stage. No significant differences were observed among the groups in (**A**) body weight or (**B**) liver weight, and no systemic side effects were noted. (**C**) Gallstone weight decreased significantly in the combination group. (**D**) Gallstone size, as measured using a 6-point grading system, decreased significantly in the UDCA and combination groups. *P < 0.05 vs. control group.

**Figure 2:**
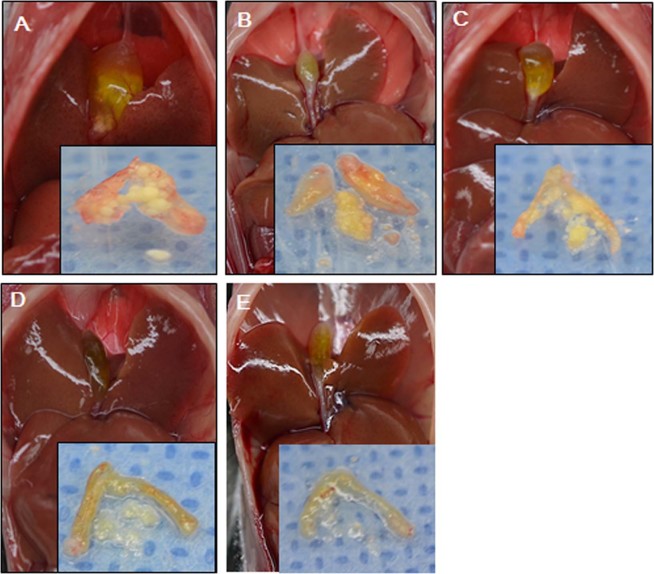
Gross findings of the liver, gallbladder, and gallstones. The photographs demonstrate the gross findings of the liver, gallbladder, and gallstones. (**A**) In the pre-treatment stage (after feeding with lithogenic diet for 8 weeks), gallstone formation was confirmed and the number and size of the gallstones were measured in 15 mice in each of the four groups (**B**–**E**). Gallstone number and size were compared between the control group (**B**) and three treatment groups ((**C**) UDCA group; (**D**) PUFA group; (**E**) combination group) during the post-treatment stage (after administering the therapeutic agent with the regular diet for 12 weeks). The number and size of the gallstones grossly reduced in all three treatment groups (UDCA, PUFA, and combination group) compared to the pre-treatment group.

There were no significant differences among groups in body or liver weight before or after treatment (Fig. 1A,B), and no systemic side effects were observed. Gallstone weight was significantly lower in the combination group than in the control group after treatment (Fig. 1C). In addition, gallstones were significantly smaller in the UDCA and combination groups than in the control group (Fig. 1D). A reduction in gallstone number and size was observed macroscopically in all treatment groups (Fig. 2A–E).

### Gallbladder histology and mucin gene expression

**Figure 3:**
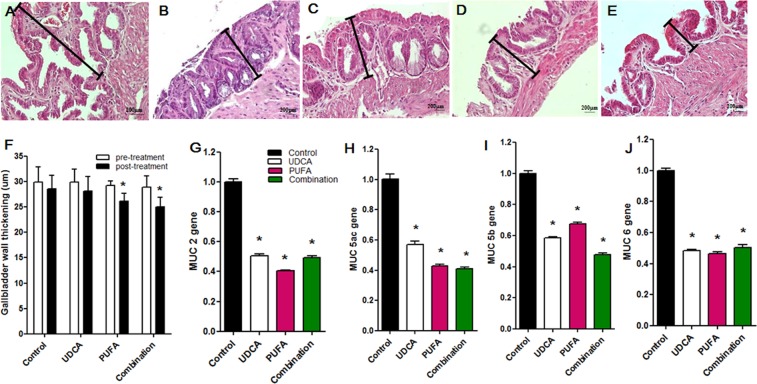
Histological mucosal changes, thickness of the gallbladder wall, and expression levels of *MUC* genes in the gallbladder. The photographs demonstrate the microscopic features of the gallbladder. (**A**) Lithogenic diet (LD) induced mucosal hypertrophy of the gallbladder in the pre-treatment stage (after feeding the lithogenic diet for 8 weeks). (**B**–**E**) Gallbladder wall thickening was compared between the control group and three treatment groups ((**B**) control group; (**C**) UDCA group; (**D**) PUFA group; and (**E**) combination group) in the post-treatment stage (after administration of the therapeutic agent with the regular diet for 12 weeks). The treatments reduced the thickness of the hypertrophied gallbladder wall. (**F**) The thickness of the gallbladder wall decreased significantly in the PUFA and combination groups. (**G**) Muc2, (**H**) Muc5ac, (**I**) Muc5b, and (**J**) Muc6 expression levels in the gallbladder decreased significantly in the UDCA, PUFA, and combination groups. *P < 0.05 vs. control group.

The thickness of the gallbladder wall was significantly reduced in the PUFA and combination groups compared with the control group (Fig. 3A–F) and was associated with a thinning of the mucosal epithelium and a reduction in the height of the gallbladder wall. The UDCA, PUFA, and combination groups showed significantly reduced expression of *Muc2*, *Muc5ac*, *Muc5b*, and *Muc6* in gallbladder tissue compared with the control group (Fig. 3G–I).

### Total phospholipids, cholesterol, and bile acid

**Figure 4:**
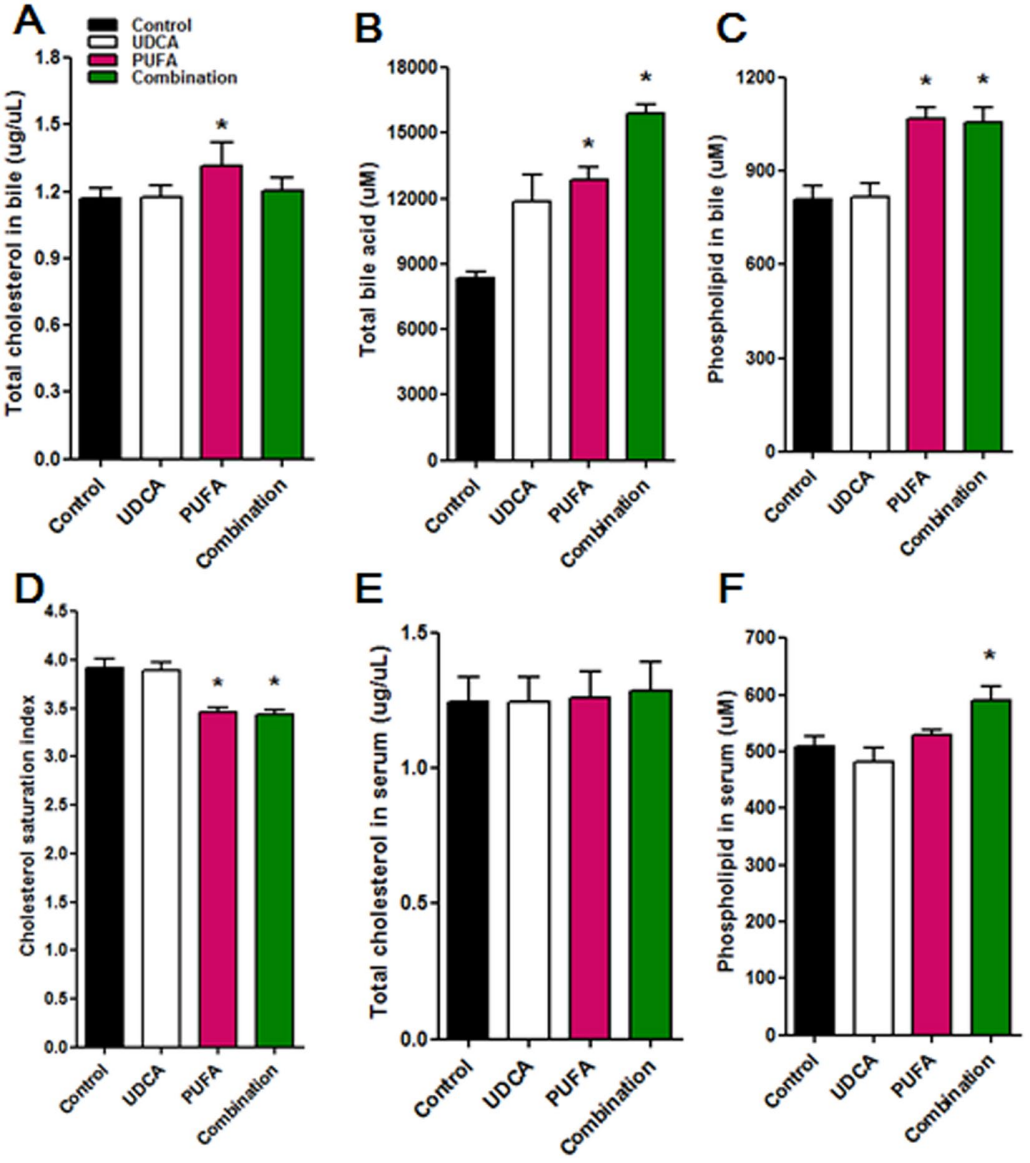
The CSI and levels of total cholesterol, phospholipids, and bile acids. (**A**) Total cholesterol level in the bile increased significantly in the PUFA group. (**B**,**C**) Both the PUFA and combination groups had significantly increased bile acid (**B**) and total phospholipid levels in bile (**C**). (**D**) As a result, the CSI decreased significantly in the PUFA and combination groups. (**E**) Total serum levels of cholesterol were similar among the groups. (**F**) Serum levels of phospholipids increased significantly in the combination group. *P < 0.05 vs. control group.

Compared with the control group, the PUFA group showed a significant increase in total cholesterol level in bile (Fig. 4A), and the PUFA and combination groups showed significant increases in levels of bile acids and phospholipids in bile (Fig. 4B,C). Consistently, the cholesterol saturation index (CSI) was significantly lower in the PUFA and combination groups than in the control group (Fig. 4D). There were no differences in total cholesterol level in serum among groups (Fig. 4E), whereas the combination group showed a significantly higher serum level of phospholipids than the other groups (Fig. 4F).

### Classes of phospholipids, cholesterol, and bile acid

**Figure 5:**
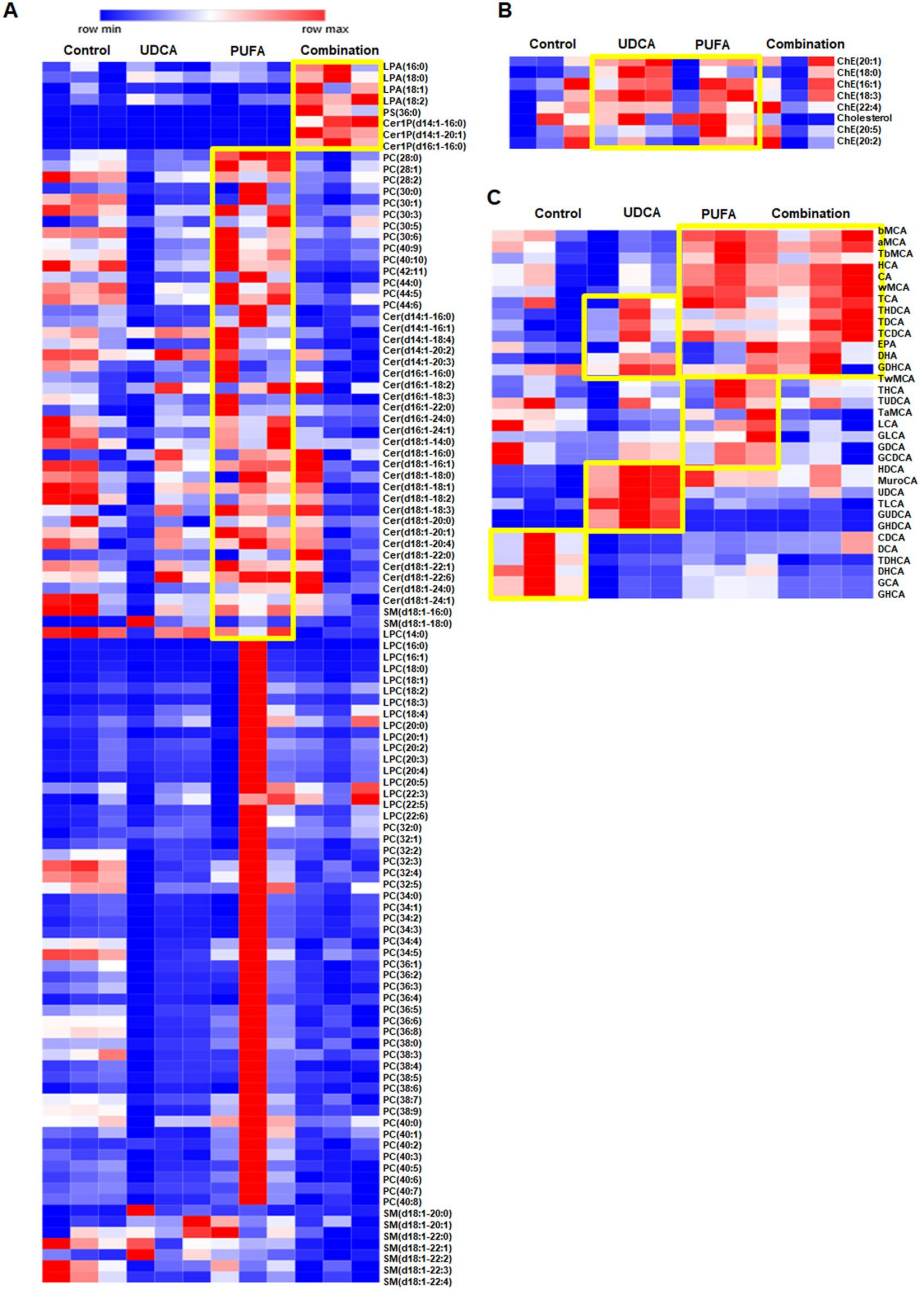
Heat maps of bile phospholipid, cholesterol, and bile acid classes. Heat map analysis of (**A**) phospholipid classes (e.g., phosphatidylcholine, lysophosphatidylcholine, lipoprotein (**a**), PS, sphingomyelin, dihydroceramide 1-phosphate, ceramide 1-phosphate), (**B**) cholesteryl ester and cholesterol, and (**C**) various bile acids. Lipid subclass profiles across the four types. Each colored four types on the map depends on a concentration value. Data in the heat map of measured by Euclidean distance using Ward clustering algorithm.

We identified 12 cholesteryl esters, 1 cholesterol, 23 ceramides, 9 sphingomyelins, 3 ceramide 1-phosphates, 50 phosphatidylcholines, 17 lysophosphatidylcholines, 4 lysophosphatidicacids, 1 phosphatidylserine (PS), 33 bile acids, and 157 lipids in bile using LC/QqQ-MS/MS. To compare levels of lipids among groups, we created heatmaps for LC-MS data. Levels of most phospholipid classes were higher in the PUFA group than in the control group (Fig. 5A). In addition, the UDCA and combination groups showed higher levels of lysophosphatidicacid and ceramide 1-phosphate species than the control group. The level of cholesterol was higher in the PUFA and UDCA groups than in the control group (Fig. 5B) but was similar between the combination and control groups.

Analysis of bile acid showed higher levels of DHA and EPA in the PUFA group and a higher level of UDCA in the UDCA group than in the control group (Fig. 5C), indicating that exogenous dosing of PUFA or UDCA resulted in increased levels of these substances. Compared with the control group, the PUFA group showed higher levels of hyodeoxycholic acid (HDCA), murocholic acid (MuroCA), UDCA, glycoursodeoxycholic acid, glycohyodeoxycholic acid, taurocholate, tauro-ω-mouse cholic acid, cholic acid, hyocholic acid, tauro-β-muricholic acid, α-muricholic acid, β-MuroCA, taurochenodeoxycholic acid, taurodeoxycholic acid, taurohyodeoxycholic acid, EPA, and DHA. The UDCA group showed higher levels of HDCA, MuroCA, UDCA, glycoursodeoxycholic acid, glycohyodeoxycholic acid, taurochenodeoxycholic acid, taurodeoxycholic acid, taurohyodeoxycholic acid, EPA, and DHA compared with the control group. The combination and PUFA groups exhibited similar increases in levels of bile acid subclasses compared with the control group.

Next, we evaluated levels of lipids in the PUFA, UDCA, and combination groups. The level of PS(36:0) was 1.5-fold higher in the PUFA and UDCA groups than in the control group (Supplementary Table 1) but was lower in the combination group than in the control group. In addition, levels of UDCA, HDCA, and MuroCA were 1.5-fold higher in the PUFA, UDCA, and combination groups than in the control group.

### Expression levels of genes related to cholesterol and bile acid production, transport, and metabolism

**Figure 6:**
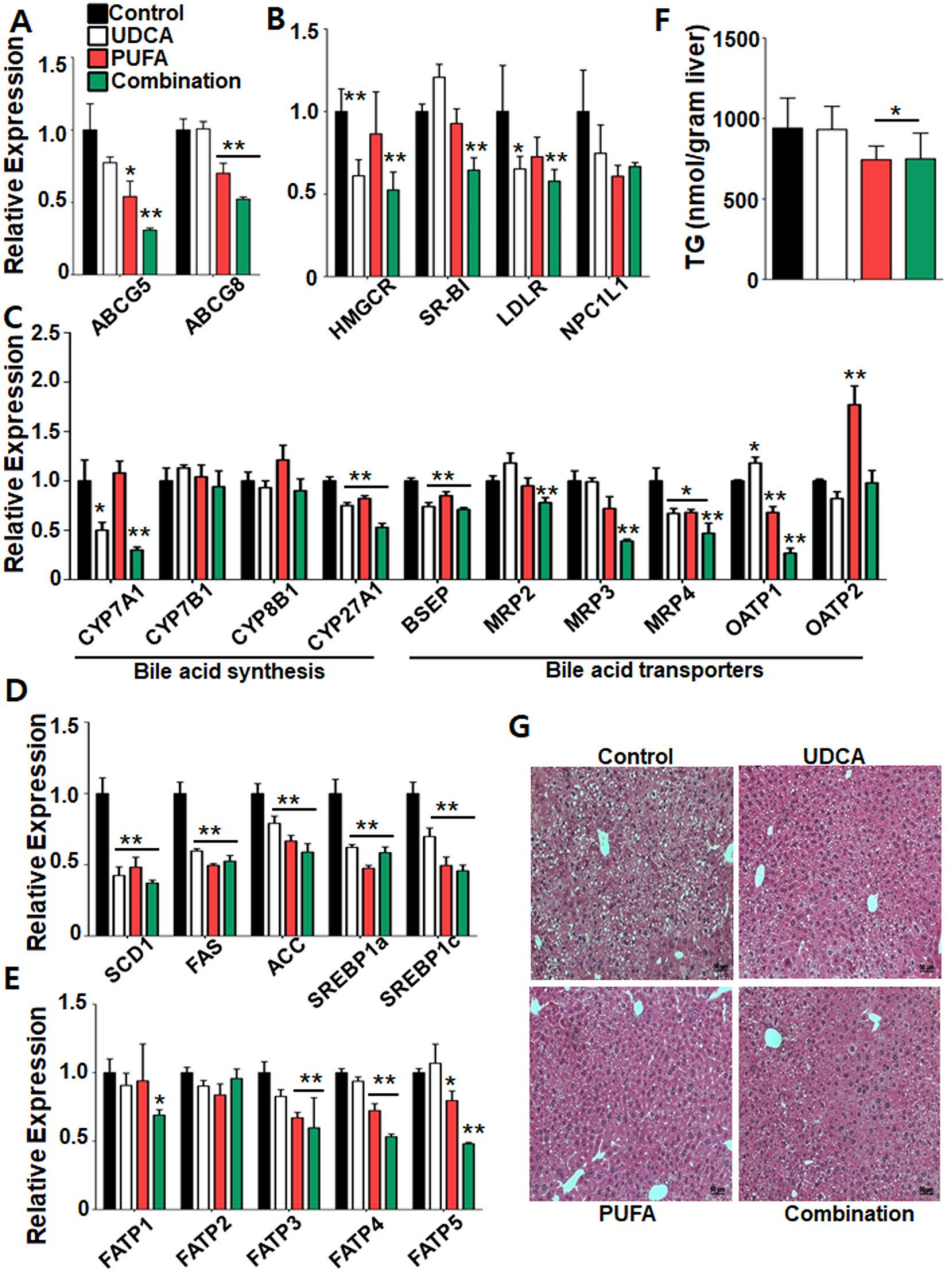
Cholesterol and bile acid production and transport, and expression levels of related genes. Hepatic expression levels of genes related to (**A**) canalicular efflux, (**B**) cholesterol synthesis, (**C**) bile acid synthesis and bile acid transporters, (**D**) lipogenesis, and (**E**) fatty acid transporters. Expression levels were normalized to that of β-actin. (**F**) Hepatic triglyceride levels. (**G**) Microscopic features of the liver. *P < 0.05 and **P < 0.01 vs. control group. Data are mean ± standard error of the mean (SEM).

To gain insight into the molecular mechanisms by which combination treatment affects gallstone formation, we analysed the expression of genes involved in cholesterol production. Cholesterol is secreted by the canalicular transporters ABCG5 and ABCG8. The PUFA and combination groups had lower expression of *Abcg5* and *Abcg8* than in the control group (Fig. 6A). Consistently, the expression of *Abcb4*, essential for efficient secretion of cholesterol from liver into bile, was also decreased in the combination group (Supplementary Fig. S2). In addition, the UDCA and combination groups had lower expression of the gene encoding HMG-CoA reductase (*Hmgcr*), the rate-limiting enzyme in cholesterol production (Fig. 6B). Although the expression level of the gene for scavenger receptor class B type I (*Sr-b1*) varied among groups, that of the gene encoding low-density lipoprotein receptor (*Ldlr*) was reduced in the UDCA and combination groups. Together, these results suggest that combination treatment modulates gallstone formation by reducing both canalicular efflux and cholesterol production.

Next, we analysed the expression levels of genes related to cholesterol metabolism that are also involved in bile acid synthesis and transport in the liver. Expression of the gene encoding cholesterol 7a-hydroxylase (*Cyp7a1*), the rate-limiting enzyme in bile acid synthesis, was reduced in the UDCA and combination groups compared with the control group (Fig. 6C). Expression of genes for 25-hydroxylcholesterol 7a-hydroxylase (*Cyp7b1*) and sterol 12a-hydroxylase (*Cyp8b1*) were unaffected by the treatments, but all three treatment groups showed reduced expression of the gene for sterol 27-hydroxylase (*Cyp27a1*).

Bile acid homeostasis is regulated by systemic bile acid signalling through bile acid transporters. The combination group exhibited significant decreases in the expression of genes encoding bile-salt exporting pump (*Bsep*), multidrug-resistance protein 2 (*Mrp2*), *Mrp3*, *Mrp4*, and organic anion-transporting polypeptide 1 (*Oatp1*) (Fig. 6C), suggesting suppression of the uptake and sinusoidal export of bile acids in the liver. Consistent with gene expression of *Bsep*, we have also noticed that a well-known nuclear bile acid receptor FXR target gene small heterodimer partner (*Nr0b2*) has been markedly reduced in the combination group, suggesting that combination treatment may reduce FXR-mediated transcriptional signalling pathway in the liver (Supplementary Fig. S3).

### Expression levels of lipogenesis and steatosis-related genes and triglyceride levels

Given our observation of reduced cholesterol production after combination treatment, we examined the expression of genes related to lipogenesis in the liver. All three treatment groups showed reduced expression of genes for stearoyl-CoA desaturase 1 (*Scd1*), fatty acid synthase (*Fas*), acetyl-CoA carboxylase (*Acc*), sterol regulatory element-binding protein 1a (*Srebp1a*), and *Srebp1c* (Fig. 6D). Next, we examined the expression of genes encoding fatty acid transport proteins. In the combination group, the expression of genes for long-chain fatty acid transport protein 1 (*Fatp1*), *Fatp3*, *Fatp4*, and *Fatp5* were decreased (Fig. 6E). These results suggest that combination treatment reduced both *de novo* lipogenesis and fatty acid uptake in the liver.

To verify these gene expression profiles, we examined triglyceride levels in the liver. Consistent with gene expression profiles, the PUFA and combination groups exhibited lower hepatic triglyceride levels than the control group (Fig. 6F). Furthermore, based on histological analyses of liver tissue, the combination group had markedly reduced hepatic steatosis compared with all other groups (Fig. 6G). Therefore, combination treatment reduces *de novo* lipogenesis and fatty acid uptake into hepatocytes, thereby decreasing the level of hepatic triglycerides. In addition to lipid metabolism, we also have noticed that proinflammatory cytokines were largely downregulated in the combination group, implying that combination treatment would have beneficial impacts to reduce hepatic inflammation (Supplementary Fig. S4).

### Transcriptome analysis of hepatic gene expression profiles

**Figure 7:**
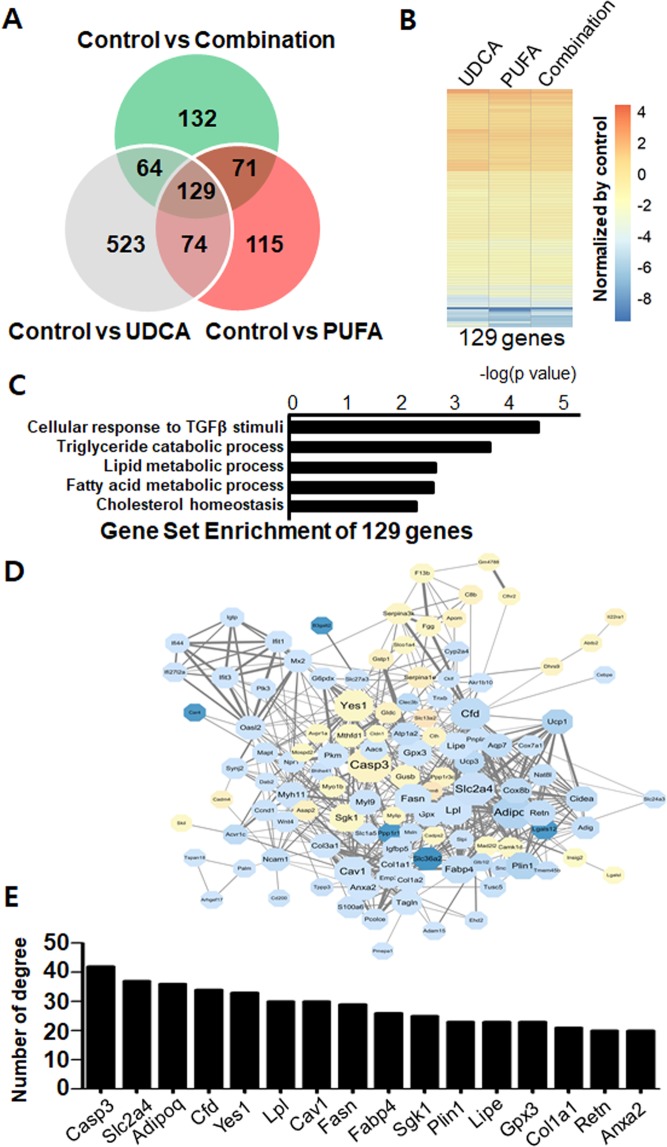
Transcriptome analyses and protein–protein interaction networks. (**A**) DEGs in the treatment groups; 129 DEGs were common across all treatments. (**B**) Heatmap of log_2_-fold changes in DEGs. (**C**) GO enrichment analyses of the 129 common target genes; bars represent enrichment scores (−log_P-value_). (**D**) Physical and functional interactions among the 129 common target genes. (**E**) Number of degrees for the main hub genes in (**A**).

To understand how the treatments affected hepatic gene expression profiles, we performed transcriptomic analyses of liver tissue by RNA-seq. The expression of 389, 523, and 396 genes was changed by PUFA, UDCA, and combination treatments, respectively (Fig. 7A). In addition, 129 genes were common targets in all treatment groups (Fig. 7B) and showed similar expression patterns across groups (Fig. 7B). GO enrichment analyses showed that several metabolic processes, including lipid metabolism and cholesterol homeostasis, were modulated by the treatments (Fig. 7C). Consistently, all treatments induced changes in metabolic pathways such as PPARγ signalling, insulin signalling, gluconeogenesis, and lipolysis regulation (Supplementary Figs 2–4). To evaluate the high-level molecular associations among these 129 genes, we used EnrichNet (i.e., network-based gene set enrichment analyses) to characterize protein-protein interaction networks (Fig. 7D). Adiponectin (*Adipo*), lipoprotein lipase (*Lpl*), *Fas*, and fatty acid binding protein 4 (*Fabp4*) were the primary hub genes (Fig. 7E), suggesting that hepatic lipid metabolism is the major target of the treatments.

## Discussion

We found that the combination of UDCA and PUFA resolved cholesterol gallstones without inducing systemic side effects, suggesting that this combination may be effective for patients with cholesterol gallstones. PUFA increased levels of phospholipids in bile, thereby lowering the CSI, and downregulated the expression of mucin genes in gallbladder tissue. This is the first study to evaluate the mechanism underlying the efficacy of treatments for gallstones using lipideomics and analysis of hepatic gene expression.

Since Wechsler and colleagues^20^ reported in 1989 that administration of omega-3 fatty acids for 6 weeks reduces biliary cholesterol levels and the CSI in adult men, several studies of the relationships among PUFA, bile, and gallstones have been performed. For example, adding fish oil to food reduces biliary phospholipid concentrations and the formation of cholesterol monohydrate crystals in prairie dogs^21^. Fish oil intake may exert these effects by enhancing the conversion of arachidonate, a biliary phospholipid, into EPA and DHA, thereby reducing biliary cholesterol saturation and suppressing cholesterol crystallization^21,22^.

Berr *et al*.^23^ reported that omega-3 fatty acids reduce biliary cholesterol saturation in humans, and Mizuguchi *et al*.^24^ showed that administration of EPA inhibits the formation of cholesterol gallstones. Thus, EPA and DHA may inhibit HMG-CoA reductase activity in the liver and increase bile secretion, thereby reducing cholesterol levels in serum and the liver^25^. Omega-3 fatty acid might not only increase biliary cholesterol secretion but also decrease the lithogenic index, as a simultaneous increase in secretion of both bile acids and phospholipids prevents cholesterol oversaturation^25^. Given these results, biliary phospholipids, in which cholesterol is soluble, could be used to treat gallstones if their molecular structure can be manipulated to increase the concentrations of phospholipids in bile, which would reduce the quantity of cholesterol crystals. In a previous study^26^, we reported that PUFA inhibits the formation of gallstones by suppressing mucin production, although we were unable to identify the underlying mechanism. Therefore, in the present study, we investigated the effects of a mixture containing UDCA and PUFA on gallstones as well as the underlying mechanism(s).

UDCA is currently used as a therapeutic agent for cholesterol gallstones. In the present study, gallstone formation tended to decrease in the UDCA group compared to the control, albeit not significantly. This lack of a therapeutic effect of UDCA alone may be attributable to the short (i.e., 12-week) administration period, as a previous study reported only a 20–30% response rate in humans who took UDCA for 2 years^16^. Similarly, the therapeutic effect in the PUFA group also failed to reach statistical significance. However, the two agents used in combination had an additive or synergistic effect on gallstone dissolution, as indicated by the significant decrease in gallstone weight and size in the combination group.

To investigate the mechanism by which combination UDCA/PUFA treatment dissolves gallstones, we measured total levels of cholesterol, phospholipids, and bile acids in bile and serum as well as the CSI, which is based on levels of cholesterol, bile acids, and lecithin in bile. The precipitation of cholesterol crystals from supersaturated bile is a prerequisite for gallstone formation^6^, whereas cholesterol is dissolved by mixed micelles of bile salts and phospholipids. Therefore, as the lithogenic index or CSI decreases, the probability of cholesterol gallstone formation also decreases^27^. As lecithin accounts for more than 95% of total phospholipids, we measured the levels of phospholipids instead of lecithin. Combination treatment did not affect the total level of bile cholesterol but significantly increased the amount of bile acids and phospholipids in bile and thus reduced the CSI. Therefore, combination treatment may dissolve gallstones by increasing levels of bile acids and phospholipids in bile, thereby altering the CSI. These changes appear to occur only in bile, as cholesterol and phospholipid levels in serum were largely unchanged.

Biliary mucin protects gallbladder epithelial cells and plays important roles in gallstone formation^28,29^. Combination treatment suppressed the expression of mucin biosynthesis genes, which affected the secretion of mucin in the gallbladder. Although mucin overproduction is a prerequisite for gallstone formation, the mechanism underlying enhanced mucin secretion during gallstone formation is unclear^30^. We suggest that combination PUFA and UDCA treatment increased levels of phospholipids and bile acid in bile, thus lowering the CSI and decreasing gallstone formation. Furthermore, combination treatment may have also reduced chemical irritation of the gallbladder mucosa, thereby decreasing the expression of mucin biosynthesis genes and mucin production and promoting nucleation in bile.

MS has potential applications in various fields, including pharmaceutical, food, and medical industries. The advantage of MS is that it can be used to perform both quantitative and qualitative analyses. It can be used to quantitatively analyse a greater variety of lipids than conventional methods, and ultra-performance LC enables separation of lipid species. In addition, multiple-reaction monitoring based on QqQ/MS allows highly sensitive analyses of target compounds. Using MS, we quantified levels of cholesterol, phospholipids, and bile acids after treatment with PUFA and UDCA. Phospholipid levels were highest in the PUFA group, and cholesterol levels were highest in the PUFA and UDCA groups, presumably due to the dissolution of gallstones. By contrast, low levels of cholesterol were observed in the combination group, suggesting rapid dissolution of gallstones and recycling of cholesterol to the liver. Furthermore, levels of most bile acid subclasses were increased in the PUFA, UDCA, and combination groups. As few studies to date have analysed bile acid subclasses, our findings provide a basis for further detailed analyses of bile acids.

Although gallstone formation is associated with metabolic syndrome, the mechanisms of this association are unclear. Here, we show that combination UDCA/PUFA treatment suppressed hepatic lipogenesis and modulated hepatic lipid metabolism, thereby alleviating hepatic steatosis and reducing gallstone formation. A previous study reports that hepatic insulin resistance is sufficient to increase biliary cholesterol secretion and promote gallstone formation^31^. Given that hepatic insulin resistance links dyslipidemia and hepatic steatosis, suppression of hepatic lipogenesis by combination treatment may prevent gallstone formation in addition to its beneficial effect on hepatic steatosis.

One limitation of this study is that we did not measure levels of biliary proteins (e.g., mucin), nucleation time, and the level of cholesterol monohydrate crystals, which means that we did not analyse the overall gallstone formation process. In addition, we did not differentiate between endogenic and exogenic bile acids in the analyses, and we collected and analysed bile from the gallbladder instead of the intrahepatic bile duct. Bile is more concentrated in the former than in the latter. In addition, we measured the total phospholipid level but could not measure that of lecithin due to the small quantity of bile collected.

In conclusion, treatment with combination of PUFA and UDCA dissolved cholesterol gallstones in mice by increasing levels of bile phospholipids and bile acids, decreasing cholesterol saturation, and suppressing mucin production without inducing systemic side effects. Our results suggest mechanisms for the therapeutic effects of PUFA on gallstones and establish a basis for future clinical trials of the therapeutic effects of the combination of PUFA and UDCA in patients with cholesterol gallstones.

## Materials and Methods

### Animals and diets

C57BL/6J (male, 8-week-old) mice were purchased from Central Laboratory Animal Inc. (Seoul, Republic of Korea). Mice (n = 160) were divided into four groups (control, UDCA, PUFA, and UDCA and PUFA combined), and 40 mice were assigned to each group. The experiment was conducted for a total of 20 weeks. A lithogenic diet (LD) was administered to all mice to induce gallstones during the pre-treatment stage for 8 weeks before the therapeutic agent was introduced. After 8 weeks, 15 mice in each group were randomly selected and sacrificed to confirm formation of gallstones and evaluate baseline characteristics during the pre-treatment stage before administration of the therapeutic agents. The mice were fasted for 12 h and anesthetized with ether using a desktop rodent anesthesia ventilator (RWD Life Science Co. Ltd., Shenzhen, China). Blood was collected from the vena cava, and bile was collected from the gallbladder. The bile collected from five mice was combined and analyzed as a single sample. Separated serum and bile samples were stored at −80 °C. The liver and gallbladder were separated and divided into two samples; one was frozen in liquid nitrogen, and the other was fixed in 10% formalin. A regular diet (RD) was administered to all remaining mice (n = 100) during the 12-week treatment stage while the therapeutic agent was administered. After the 12 weeks of treatment, the mice were sacrificed as described above.

The LD consisted of 15% anhydrous milkfat, 2.0% corn oil, 1.0% cholesterol, and 0.5% cholic acid, and contained 4,380 kcal/kg (#102136; Dyets, Inc., Bethlehem, PA, USA). The RD consisted of 6.4% crude fat (Laboratory Rodent Diets; Woojung Co., Seoul, Korea). The diets were diluted in 0.75% Tween-80 and administered daily by oral gavage. Mice in the control group were fed the RD during the treatment stage without the therapeutic agents. UDCA (12.5 mg/kg/day; Ursa®, Daewoong Pharm., Seoul, Korea), which contained 2,793 kcal/kg, was administered to mice in the UDCA group, and 51 mg/kg/day PUFA (Omacor®, Pronova Biocare, Sandefjord, Norway) was administered to mice in the PUFA group. The combined treatment group was administered a mixture of PUFA (51 mg/kg/day) and UDCA (12.5 mg/kg/day) at a 1:1 ratio.The experimental protocol and study design were approved by the Institutional Animal Care and Use Committee of Yonsei University (No. 2015-0238). All protocols were carried out in accordance with the relevant guidelines and regulations.

### Measurement of body, liver, and gallstone weight

Mouse body weight was measured weekly. After 8 weeks of consuming a LD, baseline body, liver, and gallstone weights were measured. After treatment for 12 weeks, final body, liver, and gallstone weights were measured. Gallstone size was assessed using a 6-point scoring system: 0 = clear bile; 1 = little sludge; 2 = widespread sludge; 3 = large quantity of sludge; 4 = a few small stones; 5 = several stones; and 6 = full of stones^32^.

### Hematoxylin and eosin staining of gallbladder tissue

Gallbladder tissue was fixed in 10% formalin for 2 days and sliced into six strips, which were embedded in paraffin and cut into 4-mm-thick sections. Sections were deparaffinized, rehydrated, and stained with hematoxylin and eosin. Stained sections were dehydrated, cleaned, mounted, and examined by light microscopy.

### Measurement of mucin gene expression

Total RNA was extracted from gallbladder tissue using TRIzol reagent (Invitrogen, Carlsbad, CA, USA) and treated with RNase-free DNase (Promega, Madison, WI, USA) for 30 min at 37 °C. Next, RNA was cleaned using an RNeasy Kit (Qiagen, Germantown, MD, USA). Reverse transcription reaction was performed using a commercially available high-capacity cDNA Synthesis Kit (Thermo Fisher Scientific, Rockford, IL, USA). Levels of *Muc2*, *Muc5ac*, *Muc5b*, and *Muc6* mRNA were quantified by TaqMan polymerase chain reaction (PCR) using the 18S ribosomal RNA gene (*Rn18s*) as an internal control. Data generated were analysed using Applied Biosystems 7300 software (Applied Biosystems, Foster City, CA, USA). The relative expression levels of target genes were determined using the comparative threshold cycle method. PCR primers and fluorogenic probes specific for the following genes were purchased from Applied Biosystems: *Muc2* (Mm00458299_m1), *Muc5ac* (Mm01276725_g1), *Muc5b* (Mm00466391_m1), *Muc6* (Mm00725165_m1), and *Rn18s* (Mm03928990_g1).

### Quantification of total phospholipids, cholesterol, and bile acid in bile and serum

Total phospholipid, cholesterol, and bile acid levels in bile and serum were determined using a colorimetric assay (BioAssay Systems, Hayward, CA, USA). Briefly, bile samples were diluted 10-fold and treated with working reagent. The absorbance of the samples was measured using a microplate spectrometer (Epoch, BioTek, Winooski, VT, USA) at 570 nm and 340 nm for quantification of phospholipids and cholesterol, respectively.

### Phospholipid analyses

The Folch lipid extraction method for obtaining phospholipid classes (e.g., phosphatidylcholine, lysophosphatidylcholine, ceramide 1-phosphate, cholesterol) and the bile acid extraction method were performed (Supplementary Fig. 1). All lipids were extracted with organic solvents and used a Hypersil GOLD column for liquid chromatography triple quadrupole mass spectrometry (LC/QqQ-MS/MS; Supplementary Data). All phospholipids, cholesteryl ester, and cholesterol were analyzed in positive ion mode, and bile acid was analyzed in negative ion mode.

### Data analysis and statistical modeling

Primary analysis of high-throughput sequencing data was performed with Bowtie2^33^, samtools^34^ and DESeq^35^ packages. Bowtie2 is an ultrafast and memory-efficient tool for aligning sequencing reads to long reference sequences, and samtools provides various utilities for manipulating alignments in the mapping file, including sorting, merging, and indexing. DESeq applies a statistical model to identify differential signals in RNA-seq data using a negative binomial distribution with variance and mean linked by local regression. Raw FASTQ files were generated using an Illumina NextSeq instrument (San Diego, CA, USA), and sequence reads were mapped to the UCSC mm10 reference genome and sorted using Bowtie2 and samtools. After sequence reads were mapped to the reference genome, HtSeq was used to count the reads mapped to each gene, and DESeq was used for analyses of differentially expressed genes (DEGs). Pairwise comparisons were performed between the control group and the three treatment groups to identify significant DEGs (*P* < 0.05).

### Gene ontology enrichment analyses of DEGs

Gene ontology (GO) enrichment analysis of DEGs was performed. Enriched GO terms for common DEGs in all treatment groups were identified using hypergeometric tests between DEGs and GO-annotated gene sets with a cut-off *P* = 0.05.

### Construction of protein-protein interaction networks

The STRING 9.1 network database^36^ is the one of the largest databases of direct (i.e., physical) and indirect (i.e., functional) protein-protein interactions and contains data from various sources including genomic context predictions, high-throughput experiments, co-expression analyses, and known databases. The database covers 9.6 million proteins from more than 2,031 organisms. We used this database to identify protein-protein interaction networks of the identified DEGs. As our gene sets were represented by gene symbols, the Ensembl protein identifiers in the original STRING database were converted into gene symbols using mapping information in Mouse Genome Informatics (http://www.informatics.jax.org).

### Statistical analyses

Data are expressed as mean ± standard deviation. Differences between groups were analyzed using one-way ANOVA with a post-hoc test or Mann-Whitney U-tests, as appropriate. Statistical significance was defined as *P* ≤ 0.05. Analyses were performed using SPSS (v. 18.0) software.

## Supplementary information

Dataset 1
