# Supplementary material for: Combination treatment with n-3 polyunsaturated fatty acids and ursodeoxycholic acid dissolves cholesterol gallstones in mice
Source: Sci Rep. 2019 Sep 4;9:12740. doi: 10.1038/s41598-019-49095-z (PMC6726655; doi:10.1038/s41598-019-49095-z)

Combination treatment with n-3 polyunsaturated fatty acids and ursodeoxycholic acid dissolves cholesterol gallstones in mice

Sung Ill Jang, Sungsoon Fang, Kwang Pyo Kim, Younhee Ko, Hyoseon Kim, Jieun Oh, Ga Young Hong, Su Yeon Lee, Joon Mee Kim, IL-Koo Noh, Dong Ki Lee

SUPPLEMENTARY DATA

Lipid analyses by mass spectrometry

Reagents

High-performance liquid chromatography (HPLC)-grade water, acetonitrile, methanol, chloroform, and 2-propanol were purchased from J. T. Baker (Avantor Performance Material, Inc., Radnor, PA, USA). HPLC-grade formic acid was purchased from Fluka Analytical (Sigma Aldrich, Steinheim, Germany). Lipid standards for phosphatidylcholine, lysophosphatidylcholine, sphingomyelin, phosphatidylglycerol, lipoprotein(a), ceramide 1-phosphate, phosphatidylethanolamine, lysophosphatidylethanolamine, PS, lipopolysaccharide, phosphatidic acid, lysophosphatidylinositol, phosphatidylinositol, and lysyl-phosphatidylglycerol were purchased from Avanti Polar Lipids, Inc. (Alabaster, AL, USA). The cholesteryl ester (10:0) standard was purchased from Larodan Fine Chemicals (Solna, Sweden). The cholic acid-D4, deoxycholic acid-D4, lithocholic acid-D4, glycocholic acid-D4, and glycochenodeoxycholic acid-D4 standards were purchased from Steraloids Inc., (Newport, RI, USA). EPA-D5 and DHA-D5 were purchased from Cayman Chemical (Ann Arbor, MI, USA).

Preparation of lipid samples and extraction method

Lipid standards were prepared with chloroform/methanol at a ratio of 1:1 (v/v) and were stored at –20℃. The lipid standards were diluted to their optimal concentrations before analyses. A two-step extraction method (neutral and acidic) was used to extract phospholipids and cholesterol. To extract neutral lipids, 1 mL chloroform/methanol (1:2, v/v) containing internal standards (1 µg/mL phosphatidylcholine [10:0/10:0] or lysophosphatidylcholine [13:0]; 2.5 µg/mL sphingomyelin [d18:1/12:0], phosphatidylglycerol [10:0/10:0], lipoprotein(a) [14:0], or ceramide 1-phosphate [d18:1/12:0); 5 µg/mL phosphatidylethanolamine [10:0/10:0], lysophosphatidylethanolamine [14:0], lysophosphatidylinositol [13:0], or lysyl-phosphatidylglycerol [14:0]; 12.5 µg/mL cholesteryl ester [10:0]; 25 µg/mL PS [10:0/10:0] or phosphatidylinositol [8:0/8:0]; or 50 µg/mL lipopolysaccharide [17:1] or phosphatidic acid [10:0/10:0]) was added to each sample. The sample was incubated for 3 min at room temperature and vortexed for 30 s. This process was repeated three times for a total incubation time of 10 min. The sample was centrifuged (14,000 × g) for 1 min at 4℃ and the supernatant was transferred to a new tube. To conduct the acidic extraction step, the sample was resuspended in a mixture of 750 µL methanol/chloroform/30% HCl (40:80:1, v/v/v), incubated for 5 min at room temperature, and vortexed for 30 s. This process was repeated three times for a total incubation time of 15 min. The tube was placed in ice, and 250 µL cold chloroform and 450 µL cold 30% HCl were added. The sample was vortexed for 1 min and centrifuged (6,500 × g) for 2 min at 4℃. The bottom layer was transferred to a new tube and the sample was divided into two halves and dried; one half was used for reaction with non-trimethylsilyldiazomethane (non-TMSD), and the other was reacted with TMSD. Samples for the non-TMSD reaction were dissolved in

200 µL solvent A (methanol/acetonitrile/water; 19:19:2, 20 mM ammonium formate, 0.1% formic acid) and solvent B (2-propanol, 20 mM ammonium formate, 0.1% formic acid) at a 2:1 ratio. Samples for the TMSD reaction were dissolved in 100 µL 100% methanol.

TMSD methylation

TMSD (100 µL, 2 mol L^{-1}) in hexane solution was added to the prepared sample in 100% methanol. This step was carried out in a fume hood due to the toxicity of the TMSD solution. The sample was vortexed for 30 s and incubated at 37℃ for 15 min; finally, acetic acid (20 µL) was added to quench the reaction.

Extraction of bile acids

To extract bile acids, 225 µL methanol containing bile acid internal standards (2 µg/mL cholic acid-D4, deoxycholic acid-D4, lithocholic acid-D4, glycocholic acid-D4, or glycochenodeoxycholic acid-D4) and fatty acid internal standards (20 µg/mL EPA-D5 or DHA-D5) were added to 20 µL bile samples. The mixture was vortexed for 10 s, incubated at –20℃ for 20 min, and vortexed for 30 s at 7 min intervals. After centrifugation (10,000 × g, 10 min at 4℃), the supernatant was collected and transferred to a new tube. The sample was dried in a speed vacuum concentrator and resuspended in 100 µL 50% methanol. The sample was again centrifuged (10,000 × g, 10 min at 4℃), and the supernatant was transferred to a new tube and dried in a speed vacuum concentrator.

Ultra-performance LC/QqQ-MS/MS

We used a triple quadrupole (Agilent Technologies, 6490 series) mass spectrometer coupled to a 1200-series HPLC system (Agilent Technologies, Wilmington, DE, USA) for LC-MS

analyses. A Hypersil GOLD column (2.1 × 100 mm inner diameter; 1.9 µm, Thermo Fisher Scientific) was used for lipid profiling. The temperatures of the sample tray and column oven were set to 4℃ and 40℃, respectively. The mobile phase for phospholipids and cholesterol consisted of solvent A (acetonitrile/methanol/water [19:19:2, v/v/v; 20 mM ammonium formate, 0.1% formic acid]) and solvent B (2-propanol, 20 mM ammonium formate, 0.1% formic acid) as buffers for analyses of bile acid samples. The gradient elution program was as follows (in % B): 0–5 min, 5%; 5–15 min, 5–30%; 15–22 min, 30–90%; 22–25 min, 90%; 25–26 min, 90–5%; and 26–30 min, 5%. The flow rate was 250 µL/min, and the total run time was 30 min. The sample injection volume was 3 µL for bile acid samples (analyzed in negative-ion mode) and 6 and 12 µL for non-TMSD and TMSD samples, respectively (analyzed in positive-ion mode). The analysis conditions were as follows: capillary voltage, 3,500 V in positive-ion mode and 3,000 V in negative-ion mode; sheath gas flow, 11 L/min (UHP nitrogen) at 200℃; drying gas flow, 15 L/min at 150℃; and nebulizer gas flow, 25 psi. The sheath gas and drying gas temperatures varied among the lipid species and were based on the optimal multiple-reaction-monitoring conditions.

Data processing and statistical analyses

Agilent Mass Hunter Workstation data acquisition software was used to process the LC/MS data. The B.06.00 software (Agilent Technologies) was used to export the retention time and *m/z* values of the precursor and product ions of the target lipids. We used Skyline software (MacCoss Laboratory, University of Washington, Seattle, WA, USA) to calculate the assigned peak areas of lipids in the raw data. Web-based MetaboAnalyst software (www.metaboanalyst.ca) was used to visualize extracted data.

Supplementary Table 1. Quantification of lipid species based on MS data. Significantly increased lipid species in all groups compared to the control group. (Fold change>1.5, <0.67, p-value<0.05) The DRL list commonly expressed in the group taking the omega-3, UDCA and combination. It can show the expression level of MS analysis data.

Normalized value	Control	Omega3	UDCA	Combination
PS (36:0)	1.704E-02	7.845E-03	5.275E-03	1.101E-01
UDCA	5.544E-02	1.314E-01	2.884E-01	1.831E+00
HDCA	3.508E-02	1.046E-01	1.236E-01	1.217E+00
MuroCA	3.535E-02	1.048E-01	1.237E-01	1.209E+00

Supplementary Fig 1. Schematic illustration of the workflow for phospholipid, cholesterol, and bile acid lipidomic analyses by LC-MS.

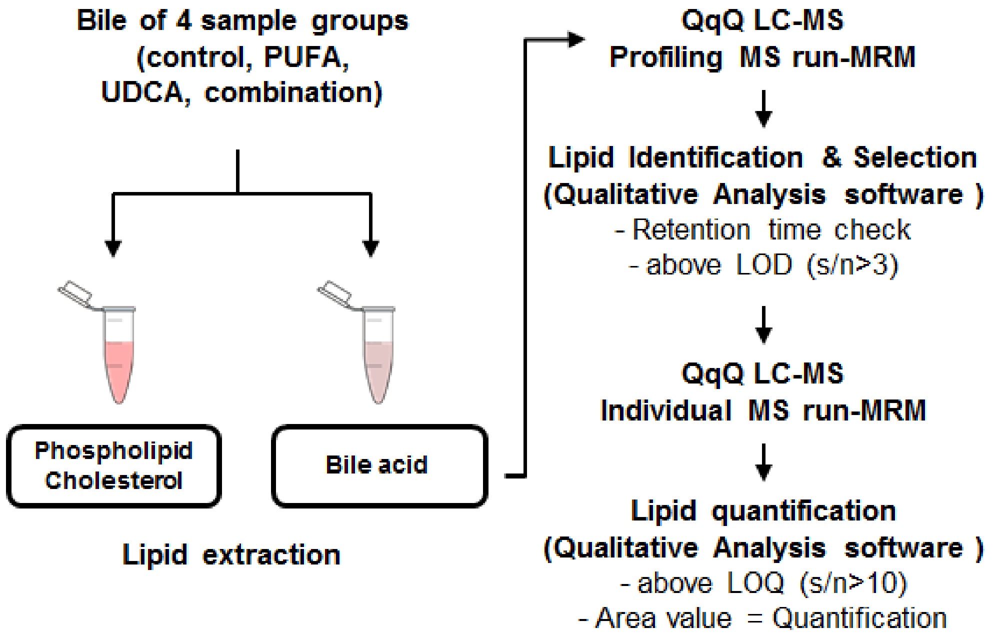

Supplementary Fig.S2. Hepatic gene expression of ABCB4. . *P < 0.05 and **P < 0.01 vs. control group. Data are shown as mean ± standard error of the mean (SEM).

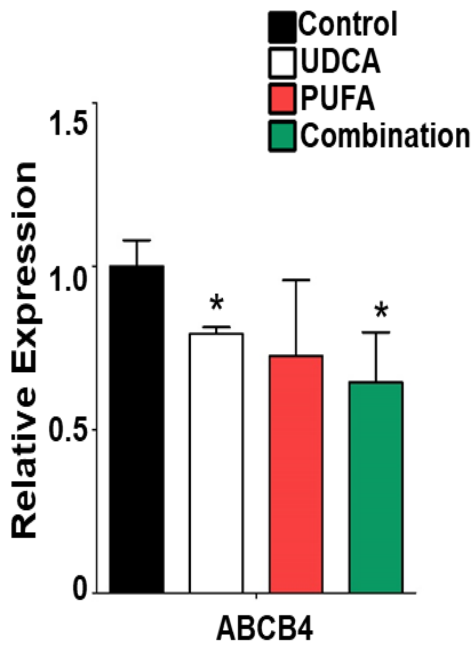

Supplementary Fig.S3. Hepatic gene expression of SHP(Nr0b2). **P < 0.05 and **P < 0.01 vs. control group. Data are shown as mean ± standard error of the mean (SEM).*

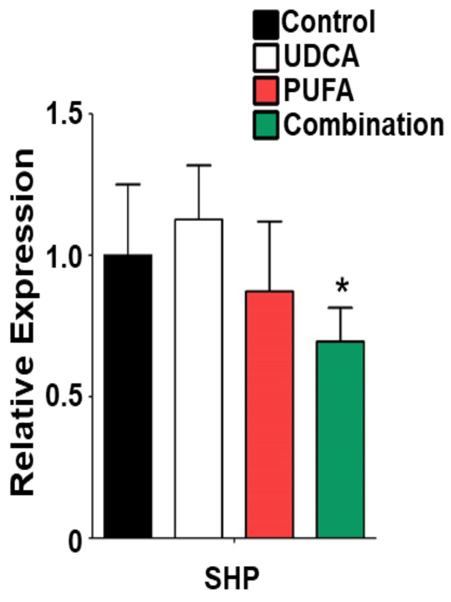

Supplementary Fig.S4. Hepatic gene expression of proinflammatory cytokines. *P < 0.05 and **P < 0.01 vs. control group. Data are shown as mean ± standard error of the mean (SEM).

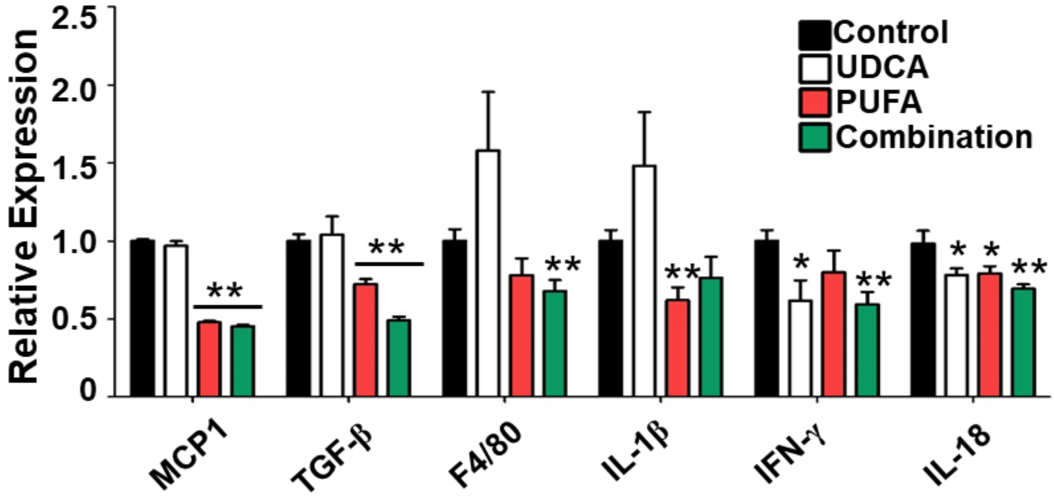

Supplementary Fig.S5. Hepatic gene expression profile in combination group normalized by control group

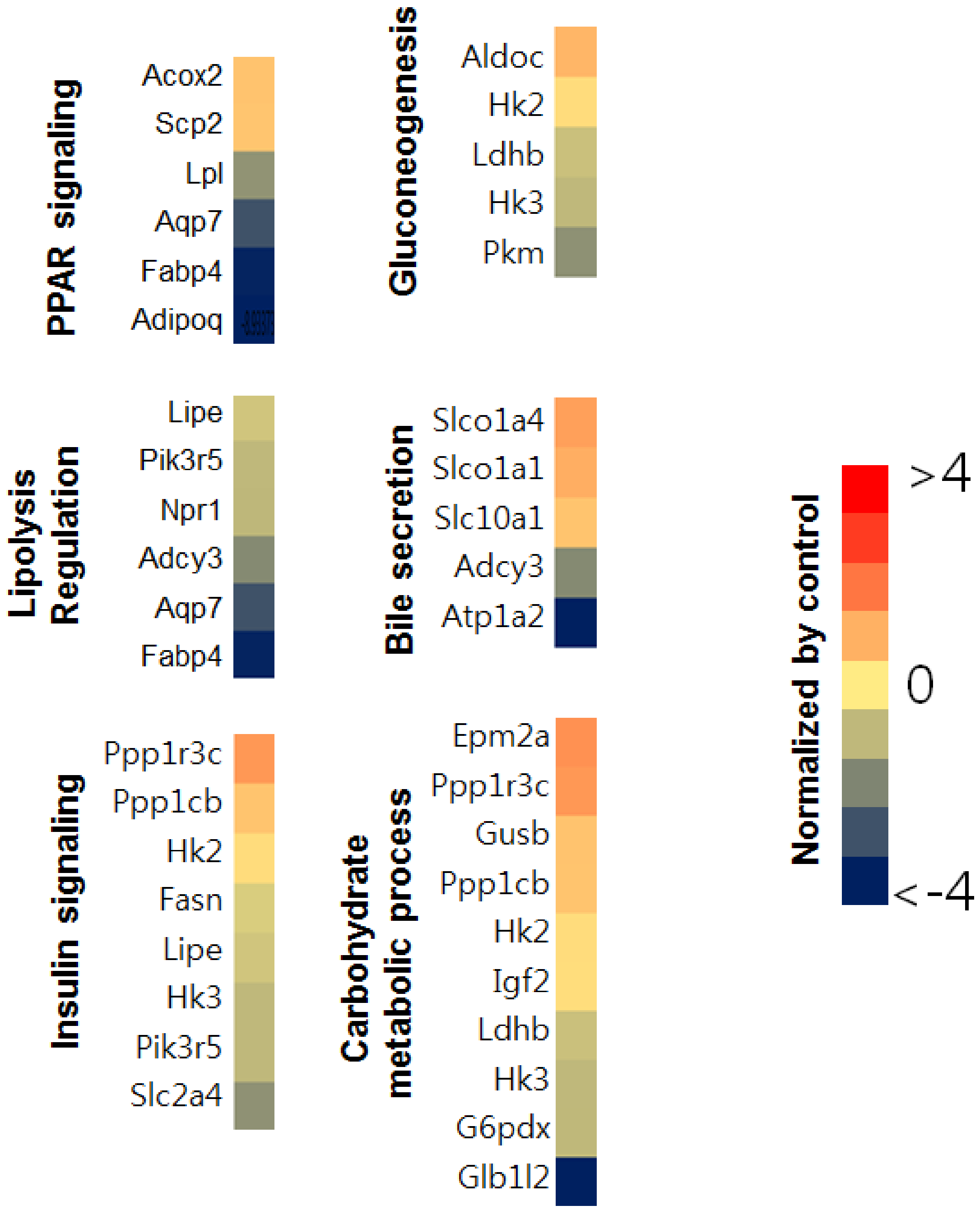

Supplementary Fig.S6. Hepatic gene expression profile in UDCA group normalized by control group

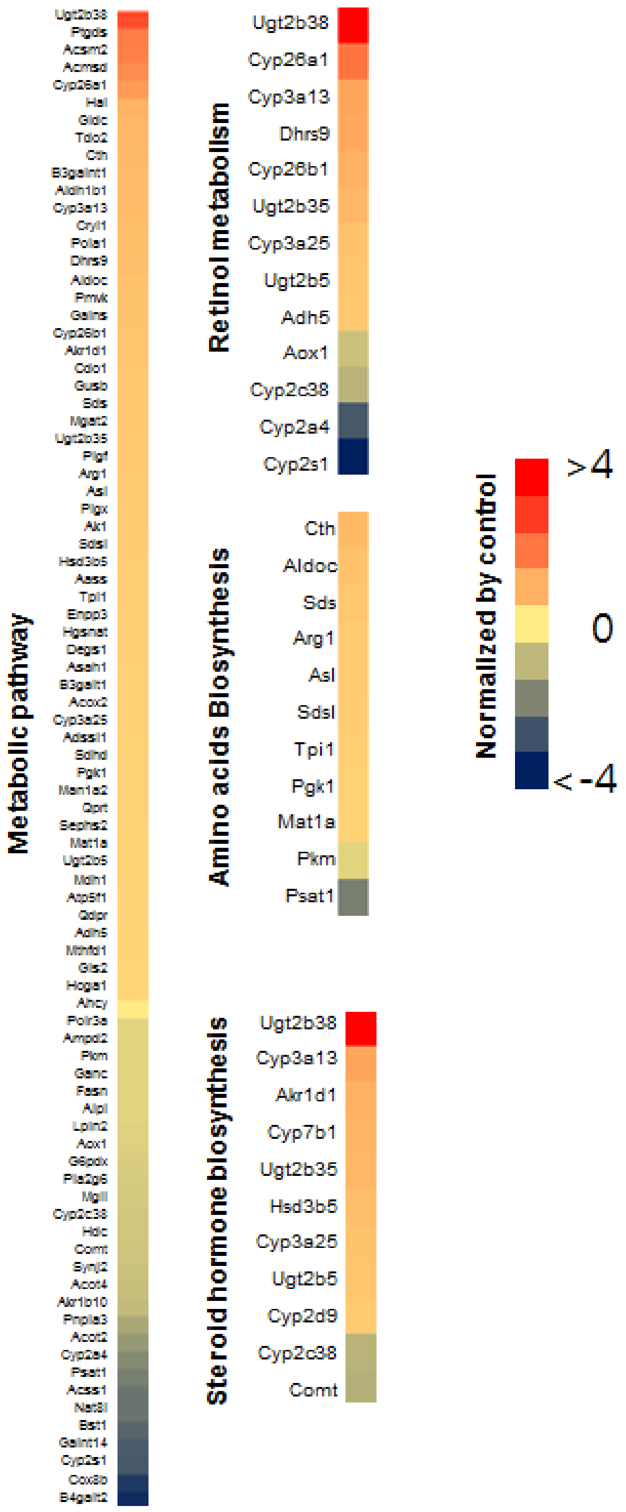

Supplementary Fig.S7. Hepatic gene expression profile in PUFA group normalized by control group

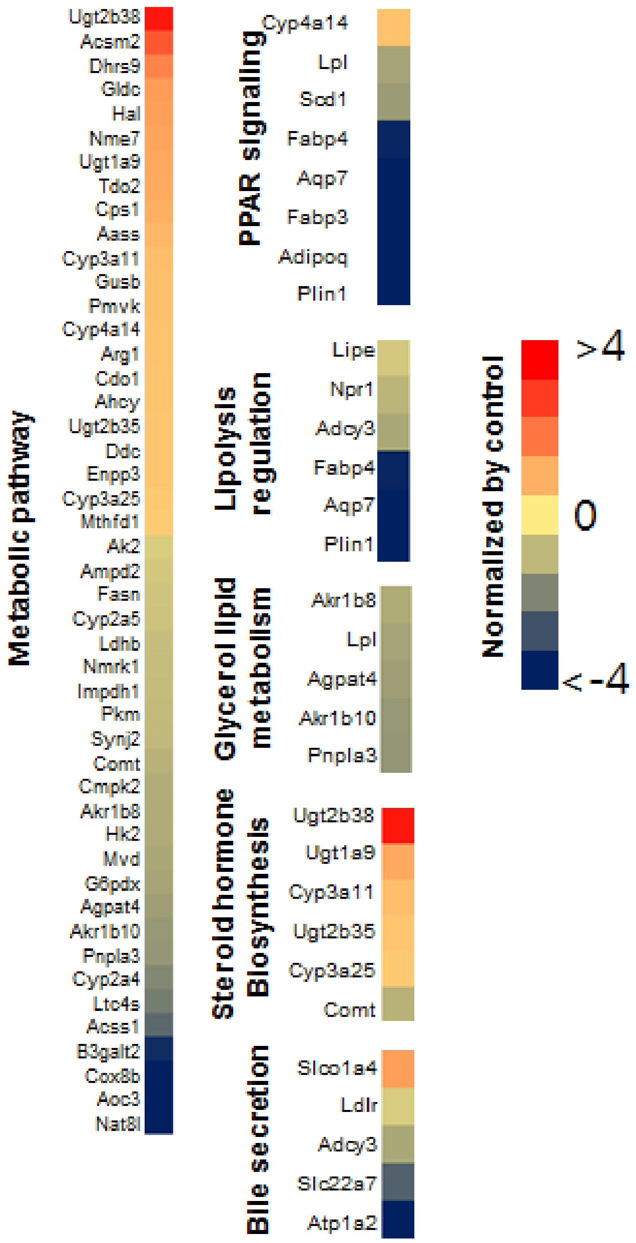

Supplement: Supplementary file 1 — Dataset 1 [file 41598_2019_49095_MOESM1_ESM.pdf]
